# Comparison of the Exposure Time Dependence of the Activities of Synthetic Ozonide Antimalarials and Dihydroartemisinin against K13 Wild-Type and Mutant Plasmodium falciparum Strains

**DOI:** 10.1128/AAC.00574-16

**Published:** 2016-07-22

**Authors:** Tuo Yang, Stanley C. Xie, Pengxing Cao, Carlo Giannangelo, James McCaw, Darren J. Creek, Susan A. Charman, Nectarios Klonis, Leann Tilley

**Affiliations:** a Department of Biochemistry and Molecular Biology, Bio21 Molecular Science and Biotechnology Institute, The University of Melbourne, Melbourne, VIC, Australia; b School of Mathematics & Statistics, The University of Melbourne, Melbourne, VIC, Australia; c Monash Institute of Pharmaceutical Sciences, Faculty of Pharmacy and Pharmaceutical Sciences, Melbourne, VIC, Australia; d Centre for Epidemiology and Biostatistics, Melbourne School of Population and Global Health, The University of Melbourne, Melbourne, VIC, Australia; e Murdoch Childrens Research Institute, Royal Children's Hospital, Parkville, VIC, Australia

## Abstract

Fully synthetic endoperoxide antimalarials, namely, OZ277 (RBx11160; also known as arterolane) and OZ439 (artefenomel), have been approved for marketing or are currently in clinical development. We undertook an analysis of the kinetics of the *in vitro* responses of Plasmodium falciparum to the new ozonide antimalarials. For these studies we used a K13 mutant (artemisinin resistant) isolate from a region in Cambodia and a genetically matched (artemisinin sensitive) K13 revertant. We used a pulsed-exposure assay format to interrogate the time dependence of the response. Because the ozonides have physicochemical properties different from those of the artemisinins, assay optimization was required to ensure that the drugs were completely removed following the pulsed exposure. Like that of artemisinins, ozonide activity requires active hemoglobin degradation. Short pulses of the ozonides were less effective than short pulses of dihydroartemisinin; however, when early-ring-stage parasites were exposed to drugs for periods relevant to their *in vivo* exposure, the ozonide antimalarials were markedly more effective.

## INTRODUCTION

*P*lasmodium falciparum caused 200 million malaria infections and 438,000 deaths in 2014 (1). While the rate is still very high, malaria deaths have in fact dropped steadily over the last decade, as countries where malaria is endemic have adopted World Health Organization (WHO)-recommended artemisinin-based combination therapies (ACTs) (2). The clinically used derivatives of artemisinin, such as artesunate and dihydroartemisinin (DHA), clear P. falciparum infections rapidly, providing prompt therapy for both uncomplicated and severe infections (3). A disadvantage of the artemisinins is their short *in vivo* half-lives (∼1 h), with the consequent need for multidose treatment regimens (3) and coadministration with a longer-lived partner drug (4).

Because malaria treatment is so heavily reliant on artemisinin-based therapies, it is extremely concerning that resistance to this drug class is now evident in six Southeast Asian countries (5, 6). Resistance is associated with mutations in the β-propeller domain of a Kelch protein, K13 (PF3D7_1343700) (7). Resistance initially manifested as delayed parasite clearance, but reports of clinical failure (recrudescence of infections) are now increasing in areas with concomitant partner drug resistance (8, 9).

Another issue with the widespread application of the artemisinins is the difficulty of maintaining the supply. The parent compound is prepared by large-scale extraction from plants, and artemisinin derivatives are generated semisynthetically, with growth, harvest, and production processes taking about 18 months (10). Recent advances in production protocols (11) and heterologous production systems (12, 13) are helping to provide the >350 million artemisinin-based treatments supplied annually (14). Nonetheless, there is an urgent need for wholly synthetic endoperoxides that are as effective and as affordable as the currently used artemisinins. Preferably, these synthetic endoperoxides will show efficacy in shorter-course treatment regimens, and ideally, they will maintain activity against artemisinin-resistant strains (10).

Artemisinins have a 1,2,4-trioxane core incorporating an endoperoxide linkage that is essential for activity (15). In the early 1990s, fully synthetic symmetrical dispiro-1,2,4,5-tetraoxane compounds with promising antimalarial activity were generated (16). Further medicinal chemistry efforts revealed that the antimalarial activity is maximized when the steric environment of the peroxide bond is carefully controlled. Asymmetrical 1,2,4-trioxolanes, in which one side of the ozonide heterocycle is sterically hindered and the other is more accessible, exhibited excellent antimalarial activity along with good *in vivo* exposure, and they were developed for clinical use (17–20).

OZ277 (arterolane maleate; also called RBx11160) was the first synthetic ozonide to undergo clinical trials and is now marketed by Ranbaxy Pty. Ltd. in India (10, 21, 22). It has good activity against all asexual blood stages of P. falciparum; however, the half-life of OZ277 is only 2- to 3-fold longer than that of DHA, and plasma exposure is lower in malaria patients than in volunteers (23, 24). OZ439 (artefenomel) appears to be more promising, exhibiting a much longer *in vivo* elimination half-life (46 to 62 h) (25, 26), and is currently undergoing phase II clinical trials (10, 25). The extended exposure profile offers the possibility that it might be effective, in combination with a second agent, as a single-dose oral cure for malaria (10, 25).

As for the artemisinins, the peroxide bond is key to the antimalarial activity of the ozonides (27), consistent with the suggestion that they need to be activated by a reduced iron source in order to exert their activity. The involvement of carbon-centered radicals as the toxic species is supported by the observation that nitroxide radical spin trap compounds antagonize the activity of both artemisinins and OZ277 (28). Nonetheless, until now, it has not been demonstrated formally that the ozonides are activated via the same mechanism as that for the artemisinins, nor is it clear whether they offer improved efficacy against artemisinin-resistant parasites. We present here an analysis of the ability of ozonide antimalarials to prevent the multiplication of the laboratory strain 3D7 as well as that of a K13 mutant isolate from Cambodia and its genetically matched K13 revertant.

## MATERIALS AND METHODS

### Culture and tight synchronization of parasites.

3D7, Cam3.II, and Cam3.II_rev parasite-infected red blood cells (RBCs) were cultured in complete medium containing RPMI 1640, 25 mM HEPES, pH 7.4, 2 g/liter sodium bicarbonate, 4 mM l-glutamine, 0.2% (wt/wt) d-glucose, 22 μg/ml gentamicin, and 0.5 mM hypoxanthine and supplemented with 5% (vol/vol) human serum and 0.25% AlbuMAX II. Cultures were incubated at 37°C in 1% O_2_, 5% CO_2_, and 94% N_2_ (29).

To generate tightly synchronized parasites, cultures were presynchronized to a 5- to 10-h window with two sorbitol treatments, and schizonts were harvested using a 70% Percoll cushion (30) when the ring:schizont ratio was about 2:1 or when the flux of ring formation was judged to be sufficient for tight synchronization (31). The harvested schizonts were added to precultured RBCs (5% hematocrit) and incubated for 0.5 to 3 h on a shaker to maximize the generation of singly infected rings (32). Excess schizonts were removed by sorbitol treatment, and the tightly synchronized ring culture was adjusted to 1 to 2% parasitemia and incubated on a shaker to achieve the parasite age postinvasion needed for the drug pulse assay. The harvested schizonts were added to precultured RBCs (5% hematocrit) and incubated for 0.5 to 1.5 h on a shaker to maximize the generation of singly infected rings.

### Drug pulse assays, interaction assays, and washing protocols.

Drugs were serially diluted in complete medium, with or without 0.2% uninfected RBCs, in V-bottomed 96-well microplates. Culture was added to the drugs (0.2% final hematocrit, 1 to 2% final parasitemia) and incubated as required before washing four times with 200 μl of complete medium, with or without transferring the cultures to wells of a new plate (after the first wash). Unwashed samples containing drugs at >10× the 50% lethal concentration (LC_50_) (standard extended-exposure assay) acted as controls for 100% parasite killing (Pt_kill_). Parasitemia was also monitored in samples with no drug (Pt_control_). Serial dilution factors were chosen in an effort to enable measurement of the minimum viability at high drug concentrations while giving good coverage of the concentration range resulting in 50% of the maximum killing effect. All assays were performed as technical duplicates.

Parasite viability was determined by measuring parasitemia in the cycle following drug treatment by flow cytometry using SYTO 61 (33). The culture medium was removed, and the cell pellets were resuspended in 20 μl SYTO 61 (2 μM in phosphate-buffered saline [PBS]), incubated for 15 min at room temperature, diluted to 200 μl, and incubated for 0.5 to 2 h before being measured. Viability was defined as the fraction of the parasite population that survived drug exposure and was able to multiply in the cycle following exposure to the drug with the same efficiency as that of the control. It is possible that some parasites enter a dormant state where they remain “viable” but unable to replicate within the period (>48 h) examined. Therefore, we used the term “viability (replication competence)” (*V*) to indicate that replication competence in the second cycle was measured. *V* was calculated using the measured parasitemias in the presence of zero drug (Pt_control_) and in the presence of >10× the LC_50_ (Pt_kill_), as follows: *V* = (Pt − Pt_kill_)/(Pt_control_ − Pt_kill_).

Nonlinear regression models were fitted to the data in Microsoft Excel by using the Solver add-in (34). LC_50_ values correspond to the drug concentrations producing a 50% loss of parasite viability in a particular assay and were determined by fitting a simple sigmoidal function to the data.

For studies of drug activation, tightly synchronized parasites (1 to 1.5% parasitemia) were preincubated in the absence or presence of sublethal concentrations of BiPy (Sigma-Aldrich) or E64d (Sigma-Aldrich) for 1 h. Serial dilutions of DHA, OZ277, and OZ439 were prepared in separate plates and added to plates containing the preincubated parasites.

### Drug stability assays.

DHA, OZ277, and OZ439 (1 μM) were incubated for different times under the conditions used for standard assays in our laboratory (wells containing 200 μl RBCs at 0.2% hematocrit in V-bottomed plates) (31). The amount of drug activity remaining in the supernatant was determined by incubating serial dilutions of the supernatant with a parasite culture for >48 h under standard assay conditions before determinations of parasitemia and viability. The relative drug concentration was estimated from the supernatant dilution producing 50% viability, with reference to a parallel assay established using fresh drug.

## RESULTS

### A modified washing procedure is required to assess ozonide potency in pulsed-exposure assays.

We previously examined the responses of laboratory and field strains of P. falciparum to artemisinins by using drug assays designed to mimic *in vivo* exposure (31, 34). In this assay format, parasites are subjected to short drug pulses, and viability (replication competence) is monitored in the next life cycle. Parasites exhibit complex drug responses that depend on their age postinvasion, their genotype, and the duration of the drug exposure (34, 35).

In this work, we examined the responses of parasites to OZ277 and OZ439 pulses of different durations. Because the physicochemical properties of the ozonides are different from those of DHA, it was important to ensure complete removal of the drugs after the drug pulse to ensure that the activity was not overestimated. That is, the wash procedure needed to be sufficiently stringent to ensure that the drug was diluted to a level well below the inhibitory level.

Our standard protocol for performing pulse assays involves incubating cultures (200 μl; 0.2% hematocrit) with drugs in V-bottomed microplates for a specified period and then removing the drug by washing the cells four times with complete culture medium (containing 5% serum and 0.25% AlbuMax II) (31). In this procedure, the infected RBCs are maintained within the same microplate wells during the course of the assay. This approach is sufficient to dilute the concentration of highly soluble drugs present in the supernatant >10,000-fold. To test the efficiency of this procedure in removing the ozonides, suspensions of uninfected RBCs (0.1% hematocrit) were incubated for 3 h in the presence of drugs at concentrations of up to 2 μM, and the RBCs were washed using the standard protocol. In order to detect any drug activity remaining in the wells, we added aliquots of RBCs infected with the laboratory parasite 3D7 (2 to 4% parasitemia) to the washed uninfected RBCs and measured the parasitemia after culture for >48 h to allow the parasites to enter the next parasite cycle.

**FIG 1:**
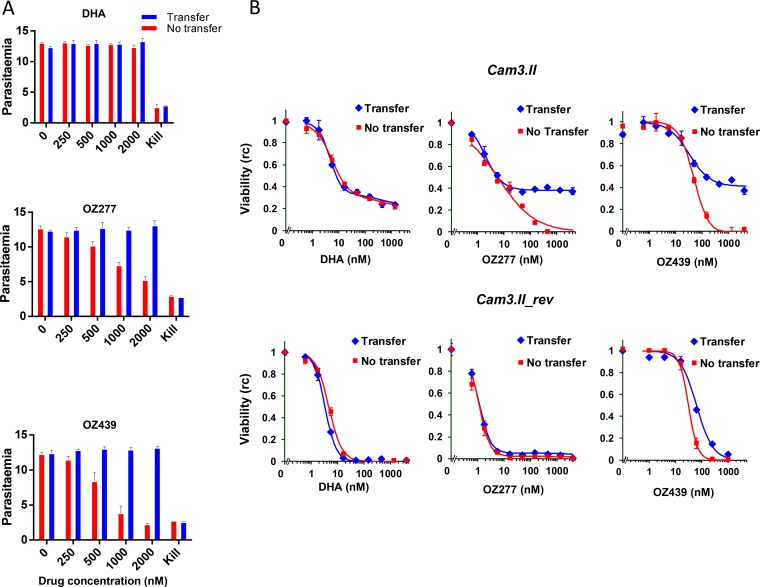
Enhanced washing protocols are required for pulse assays of ozonide antimalarials. (A) The indicated concentrations of endoperoxides were incubated with RBCs (0.2% hematocrit) under standard culture conditions (complete medium, 37°C, V-bottomed microplate) for 3 h. The RBCs were washed using the standard protocol, with the cells maintained in their original well (red bars) or transferred to a fresh well (blue bars) prior to the addition of 200 μl of parasite-containing culture (0.1% hematocrit, 2 to 4% parasitemia, strain 3D7). The samples were then cultured for >48 h prior to determining viability (replication competence). Data represent the averages for two (OZ439) or one (DHA and OZ277) experiment, each with ≥2 technical replicates. (B) Early-ring-stage K13 mutant (Cam3.II) or revertant (Cam3.II_rev) cultures were incubated for 6 h with the indicated drugs. During a 4-step washing protocol, the cultures were maintained in their original wells (red symbols) or transferred to fresh wells (blue symbols). The samples were then cultured for >48 h prior to determination of viability (replication competence [rc]). Data represent a typical experiment of two independent experiments, each performed in duplicate.

We found that DHA at concentrations of up to 2 μM was efficiently removed by the washing procedure, such that the parasitemia was unaffected (Fig. 1A, top panel). In contrast, wells incubated with ≥500 nM ozonides retained significant drug activity after the standard washing protocol, producing a reduction in the parasitemia during the postwash incubation period (Fig. 1A). For OZ439, 50% of parasites were rendered nonviable when the initial added concentration was between 500 and 1,000 nM. Given that the LC_50_ for exposure times of >48 h (LC_50_ >48 h_) for OZ439 is ∼9 nM (Table 1), this suggests that ∼1% of the added OZ439 remained associated with the well. In contrast, when we modified the washing procedure so that the uninfected RBCs were transferred to fresh microplate wells after the first wash, there was little or no drug activity remaining (Fig. 1A). These results indicate that transferring the samples to fresh wells substantially improves the efficiency of washing.

**TABLE 1:** Potencies of DHA and ozonides against different P. falciparum strains in standard assays (constant drug exposure for >48 h)

Strain	LC_50>48 h_ (nM)^*a*^		
	DHA	OZ277	OZ439
3D7	2.6 ± 0.8	1.8 ± 0.7	8.7 ± 1.4
Cam3.II	2.3 ± 0.4	1.0	4.4 ± 0.7
Cam3.II_rev	2.4 ± 0.2	1.3 ± 0.1	6.1 ± 1.4

a Data are means ± standard deviations (*n* = 3).

Inefficient drug removal compromises the accuracy of determinations of drug potency, particularly under conditions where the concentration producing a 50% loss of viability is high, as in the case of the very early ring stage of artemisinin-resistant parasites from the field. To test this, we examined different washing protocols by using a laboratory-adapted field isolate from Cambodia (Cam3.II; K13 mutant) that was previously shown to exhibit reduced sensitivity to DHA (35, 36). We also employed a reverted transfectant in the same line (Cam3.II_rev), in which the *K13* wild-type genotype has been restored (36).

We added drugs at the least sensitive (very early ring) stage of the K13 mutant for a period of 6 h, washed the cultures, and examined viability after >48 h (Fig. 1B, top panels). The dose-response profile obtained with DHA was unaffected by the washing protocol, with both the standard and enhanced washing methods associated with residual viability values of about 30% at physiologically relevant drug doses (∼700 nM) (Fig. 1B, top left panel; see Table S1 in the supplemental material). This failure to render all of the parasites nonviable, even at high drug concentrations, confirms the resistance phenotype, while the overlapping profiles show that DHA is efficiently diluted using the standard washing protocol. In contrast, the loss of viability following pulsed exposure to the ozonides was substantively dependent on the washing regimen. The standard washing regimen resulted in an almost complete loss of parasite viability (>95%) by OZ439 and OZ277 at the highest concentrations examined (Fig. 1B, top panels). In contrast, the residual viability was much higher when the transfer washing regimen was employed (∼40% residual viability) (Fig. 1B, top panels). The problem was less obvious for the sensitive very early ring stage of the K13 revertant (Cam3.11_rev) (Fig. 1B, bottom panels), because the LC_50_ values were much lower and inefficient washing was thus more difficult to detect.

Because OZ439 is lipophilic, there is the possibility that a substantial fraction of the added drug is associated with cell membranes and the walls of the wells during the initial incubation period. This would mean that the added concentration of ozonides would not reflect the concentration in the medium, which would influence the ability to compare the *in vitro* efficacies of different drugs and to extrapolate to an *in vivo* setting. We were therefore interested in determining whether OZ439 activity in the culture supernatant was significantly decreased as a result of binding to surfaces. To estimate the proportion of the drug activity that remains in the supernatant, we mixed different concentrations of OZ439 with uninfected RBCs in wells, recovered the supernatants, and serially diluted them in new wells, with the addition of fresh 3D7-infected RBCs (0.2% hematocrit, 1 to 2% parasitemia). The drug activities remaining in the supernatant, determined with reference to equivalent freshly prepared samples of OZ439, were found to be ∼90% of the added concentration over a broad concentration range (see Fig. S1 in the supplemental material). This demonstrates that under the conditions of our assays, ∼90% of the added drug is retained in the culture supernatant.

**FIG 2:**
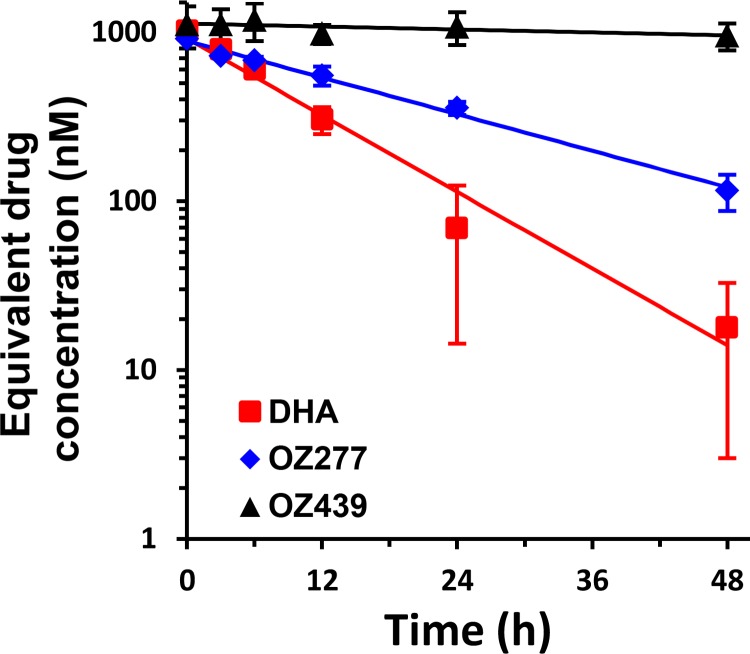
Degradation of drugs under culture conditions. Drugs (1 μM) were incubated for different times under our standard conditions for drug assays (200 μl of RBCs at 0.2% hematocrit in V-bottomed plates), after which the supernatant was collected and transferred to a new well containing infected RBCs. Parasite viability (replication competence) was determined after >48 h and compared with the viability of parasites exposed to equivalent freshly prepared drug dilutions. The drug dilution required to render 50% of parasites nonviable was determined and used to estimate the effective drug concentration remaining in the supernatant. Data represent the averages for two independent experiments, with error bars representing the ranges for duplicates. Lines correspond to best fits, with half-lives of 8, 17, and 210 h for DHA, OZ277, and OZ439, respectively.

Another possible source of error in estimates of drug efficacy may arise due to degradation of the endoperoxide during the incubation pulse. To estimate degradation losses, we incubated drugs (1 μM) with uninfected RBCs under our culture conditions for different periods before serially diluting the supernatants, with the addition of fresh infected RBCs, and examining parasite viability after >48 h. The effective drug activity remaining was determined with reference to freshly prepared drugs by determining the drug dilution required to render 50% of the parasites nonviable. DHA lost activity with a half-life of ∼8 h (Fig. 2). OZ277 lost activity more slowly, with a half-life of ∼17 h. In contrast, OZ439 was stable under culture conditions, with a half-life of ≫48 h and <20% loss after 48 h (Fig. 2). The observed difference in the stabilities of OZ277 and OZ439 is broadly consistent with previously reported differences in stability in blood (19).

### Exposure time dependence of the response of parasites to ozonides.

**FIG 3:**
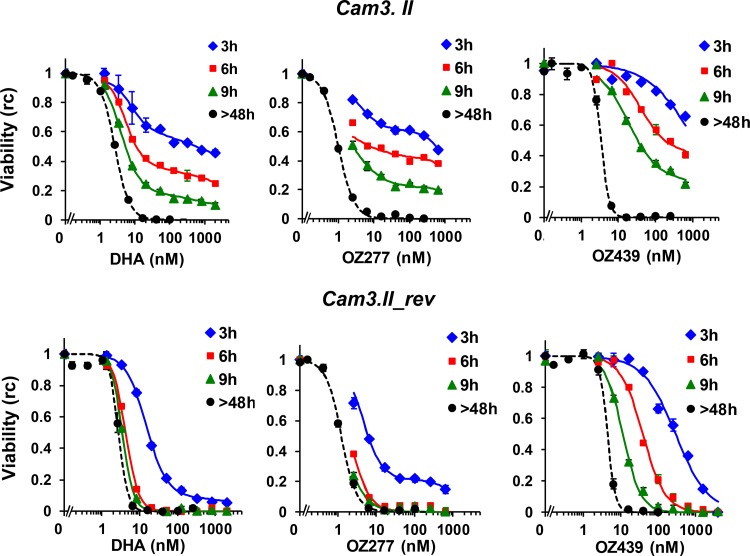
Effects of pulse duration of different endoperoxides on viability (replication competence) of K13 mutant and revertant parasites. Very-early-ring-stage Cam3.II (top) and Cam3.II_rev (bottom) parasites were incubated with drugs for 3, 6, and 9 h (blue, red, and green symbols, respectively), or the drug pressure was maintained for >48 h (black symbols). For pulse assays, the drugs were removed using the enhanced washing protocols, and parasite viability (rc) was determined after >48 h. Data shown are representative of experimental replicates (*n* = 2 for pulse assays and *n* = 3 for >48-h assays). Error bars correspond to the ranges for technical replicates from the individual experiments. Solid curves correspond to the best fits obtained with the CED model, using the parameters for replicate 1 from Table 2. The dashed curves are included as a visual aid to illustrate the dose-response profiles of parasites exposed to drugs for >48 h.

We initially compared the activities of the ozonides and DHA against the K13 mutant and revertant parasites and against the laboratory strain 3D7 by using a “standard” assay format where ring-stage parasites (∼20-h synchronization window) are left in contact with drug for more than one parasite life cycle (>48 h) before assessment of parasitemia. With extended exposure, the parasites exhibited very similar LC_50_ values for all three drugs (1 to 9 nM) (Table 1 and Fig. 3), in agreement with previous work (17, 19). However, we previously showed that drug activity determined using a >48-h exposure is a poor predictor of the *in vivo* efficacy of artemisinins, as these drugs exhibit different (usually much shorter) exposure times *in vivo* (34, 35). We therefore used our improved washing protocols to compare the exposure time dependence of the parasite responses, focusing on the stages where parasites exhibit relatively low sensitivity.

We found that the very early ring stage of the Cam3.II strain showed markedly decreased sensitivity to a 3-h pulse of DHA compared with the Cam3.II_rev strain (Fig. 3A, left panels; see Table S1 in the supplemental material), as reported previously for other K13 mutant strains (35). In particular, >50% of Cam3.II parasites survived a 3-h 700 nM DHA pulse, compared to ∼5% of Cam3.II_rev parasites. Similarly, the K13 mutant exhibited significantly lower sensitivities to 3-h and 6-h pulses of OZ277 and OZ439 (Fig. 3, top panels; see Table S1). It is important that the decreased efficacy (in a 6-h exposure) was less evident when the standard (no transfer) washing procedure was employed (Fig. 1). This emphasizes the need for particular care in pulse assays of the efficacy of ozonides.

We also examined the mid-ring stage of 3D7 parasites, which we previously showed to exhibit relatively low sensitivity to artemisinins (34). In agreement with our previous report, the mid-ring stage showed low sensitivity to a 1.5-h pulse exposure to DHA (LC_50_3 h_, 180 nM) but exhibited a marked increase in sensitivity at longer exposure times (see Fig. S2 in the supplemental material). We found that mid-ring-stage 3D7 parasites exhibited an even lower sensitivity to 1.5-h pulsed exposures to OZ277 and OZ439 (LC_50_1.5 h_ > 1,000 nM). Again, the parasites became sensitive to the ozonides as the exposure time was increased (see Fig. S2). When treatment was initiated at the trophozoite stage, the exposure time dependence of drug action was less dramatic but was still apparent (see Fig. S2).

The complex dependence of drug activity on different exposure factors makes it difficult to directly quantitate or compare the activities of different endoperoxides. We previously developed a cumulative effective dose (CED) model that is able to describe the complex concentration and exposure time dependence of artemisinin-mediated loss of viability and that facilitates comparisons of different drugs and their effects on different strains (34). According to this model, the extent to which a particular endoperoxide renders parasites nonviable (replication incompetent) is dependent on its “effective dose” (likely representing the amount of activated endoperoxide) rather than the administered concentration (37). The effective dose is a saturable function of the administered drug concentration, with the parameter *K_M_* representing the drug concentration that produces half the maximum effect (34). The minimum time required to render 50% of the parasites nonviable at saturating drug concentrations (*t*^_50_) and the slope of the sigmoidal function that relates the effective dose to the parasite viability (γ) are also determined through application of the CED model to viability data. In performing this analysis, we do not explicitly take into account the changing drug concentrations over time (as revealed in Fig. 2), as the CED model can characterize only the cumulative effect on viability over an entire drug pulse, independently of any detailed changes in kinetics during that drug pulse. In the case of OZ277 and OZ439, this does not have a major influence on the analysis (i.e., parameter estimation), as the experimental observations occur over a period (9 h) much shorter than the *in vitro* half-lives of the drugs (Fig. 2). In the case of DHA (with a half-life of 8 h), drug instability will lead to an overestimation of *K_M_* values and will also influence the underlying slope of the dose-response curve (γ) but will not affect the magnitude of *t*^_50_.

The CED model is able to describe the observed dependence of drug activity against early-ring-stage Cam3.II and Cam3.II_rev parasites on drug concentration and exposure time (Fig. 3, fitted solid lines [these provide good fits to the 3-, 6-, and 9-h exposure data]). Analysis of the fit parameters showed that the *K_M_* value is essentially independent of the strain but shows a more pronounced dependence on the identity of the drug (Table 2). It is interesting that the average *K_M_* value obtained for DHA treatment of the K13 revertant and mutant strains used in this work (mean ± standard error of the mean [SEM], 15 ± 2; *n* = 4; values were obtained by using the combined data for Cam3.II_rev and Cam3.II from Table 2) is similar to values reported earlier for another pair of wild-type and K13 mutant strains (14 and 19 nM for PL2 and PL7, respectively [35]). In contrast, the combined *K_M_* value for OZ439 (60 ± 20; *n* = 4) is markedly greater than that for DHA, while the value for OZ277 (12 ± 7; *n* = 4) is similar to that for DHA. Although the *K_M_* values determined in this analysis are macroscopic constants and are influenced by the different chemical properties of the drugs (including different levels of binding to proteins), the observed trends are broadly in agreement with their relative abilities to be activated by iron (38).

**TABLE 2:** CED model parameters describing the actions of DHA and ozonides on early ring stages of strains Cam3.II_rev and Cam3.II

Parameter	Values for R1, R2^*a*^					
	DHA		OZ277		OZ439	
	Cam3.II_rev	Cam3.II	Cam3.II_rev	Cam3.II	Cam3.II_rev	Cam3.II
*t*^_50_ (h)	1.4, 1.2	3.0, 3.3	1.4, 0.9	3.7, 4.1	2.6, 2.2	5.1, 5.2
*K_M_* (nM)	17, 18	11.5, 12.5	6, 30	4, 7	35, 100	28, 76
γ	3.2, 2.4	1.6, 1.8	2.2, 1.6	1.3, 1.5	3.2, 2.1	1.9, 2.2

a R1 and R2 correspond to the different experimental replicates.

The CED analysis shows that sensitivity to endoperoxides is largely determined by the *t*^_50_ value (34). The use of isogenic lines in the present study revealed that the K13 mutation (R539T) increased the *t*^_50_ value for DHA from ∼1.3 h to ∼3.2 h (Table 2), a trend in general agreement with that previously reported for wild-type (PL2) and K13 mutant (PL7) field strains (values of 1.4 and 4.9 h, respectively [35]). Interestingly, early-ring-stage K13 revertant parasites exhibited similar *t*^_50_ values when exposed to either OZ277 or DHA (Table 2). K13 mutant parasites exhibited larger *t*^_50_ values, but again they were similar for OZ277 and DHA. OZ439 also showed an increased *t*^_50_ value for the K13 mutant; however, in this case, the times required to render 50% of parasites nonviable (*t*^_50_ values) were 1 to 2 h longer for both strains than the value for DHA.

### Simulation of *in vivo* parasite responses.

To consider the possible clinical implications of our findings, we modeled parasite responses to drug exposure at levels that might be expected to be achieved in patients. The different *in vivo* pharmacokinetic properties of the drugs, as well as their different pharmacodynamic behaviors, preclude a simple comparison using the observed *in vitro* concentration-response profiles shown in Fig. 3. Moreover, traditional approaches for assessing drug efficacy *in vivo*, such as measuring peak concentrations and areas under the curve (AUC), are inappropriate for drugs, such as the artemisinins, which exhibit short *in vivo* half-lives and lag times in their action.

**FIG 4:**
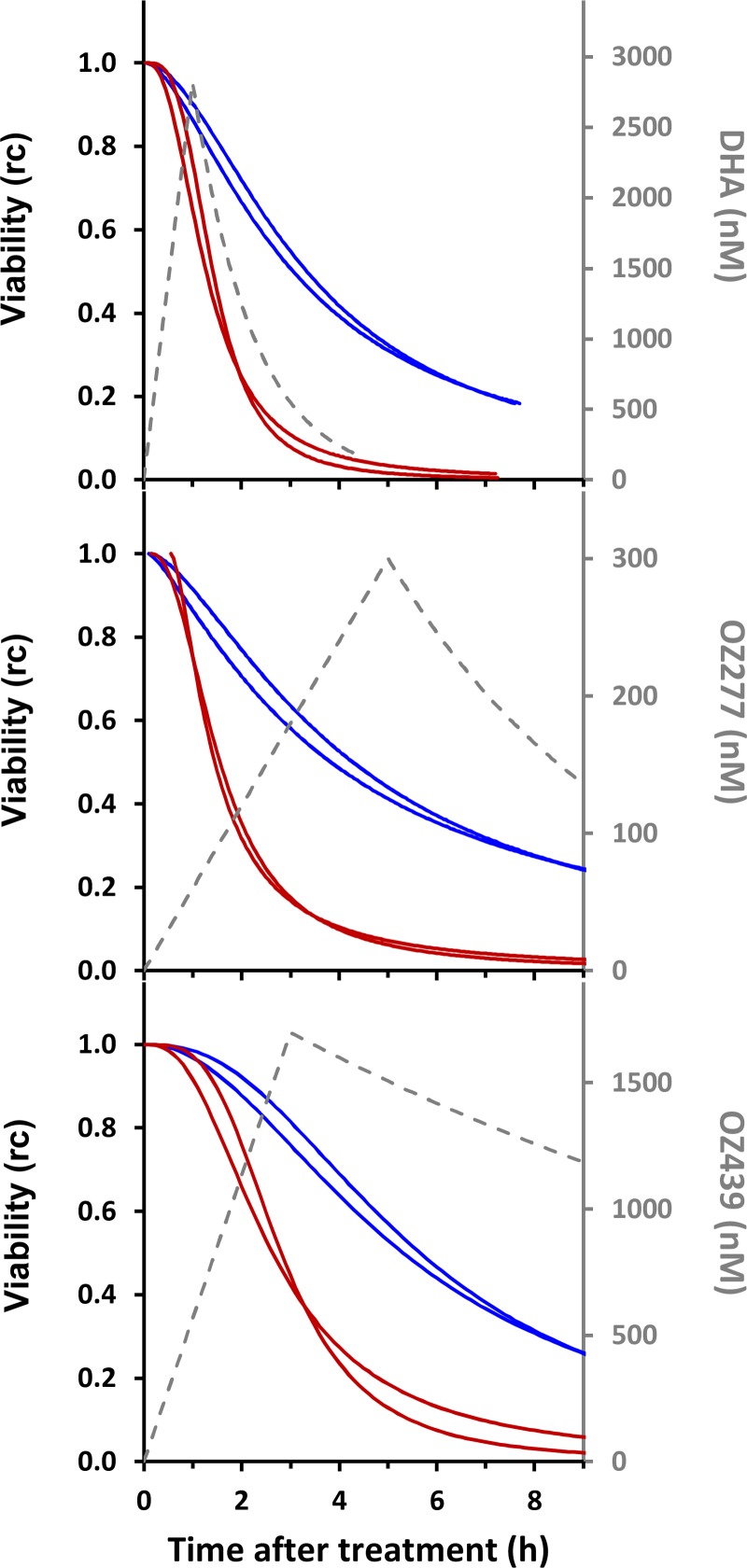
Simulation of *in vivo*-like responses to single-dose treatments with different drugs. Viability profiles for K13 revertant (Cam3.II_rev; red curves) and K13 mutant (Cam3.II; blue curves) parasites in hypothetical patients following treatment at the very early ring stage with a single dose of DHA, OZ277, or OZ439 were calculated using the CED model parameters shown in Table 2 and the pharmacokinetic parameters shown in Table S2 in the supplemental material. Viability calculations were performed at times after treatment when the *in vivo* drug concentrations were greater than the *K_M_* value, with the assumption that these represent saturating drug concentrations. (See the text for further details.) The duplicate blue and red curves correspond to independent simulations performed using CED model parameters derived from the analysis of independent experimental replicates (Table 2). The dashed gray lines correspond to estimates of the *in vivo* drug concentrations based on reported values.

In the present study, we investigated how efficiently a single dose of drug might reduce the parasite burden in an infection comprised of early-ring-stage parasites. We chose early rings because this is the stage at which K13 mutants exhibit the highest ability to survive drug treatment (7, 39). For this simulation, we took advantage of the fact that the reported pharmacokinetics of each drug (summarized in Table S2 in the supplemental material) indicate that saturating concentrations of all the drugs are achieved relatively quickly. That is, the time required (following drug administration) for drug concentrations to reach *K_M_* values is typically less than 20 min, and these saturating levels are maintained for many hours. As a result, the CED model predicts that the viability (*V*) will vary as a function of drug exposure time (*t*^*e*^) according to the equation *V*(*t*^*e*^) = [1 + (*t*^*e*^/*t*^_50_)^γ^]^−1^ while these saturating conditions are maintained. That is, early killing events will be independent of the precise pharmacological concentration. In contrast, viability at longer times will depend on both the administered drug dose and the drug pharmacokinetics (i.e., how long it takes for the drug to decay to sublethal levels). In this work, we used the CED model parameters that describe the response of early rings to exposure times of up to 9 h (Table 2) and incorporated knowledge of the pharmacokinetic profile for each drug (see Table S2) (24, 25, 40) in order to generate simulations of parasite viability *in vivo* over the first 9 h following a single-dose exposure (Fig. 4).

One consequence of the fact that saturating drug concentrations are achieved rapidly and sustained for several hours *in vivo* is that the times required to achieve a 50% reduction in viability of early rings are well approximated by the *t*^_50_ values shown in Table 2. Thus, the CED model analysis indicates that the much slower reduction of parasitemia observed for resistant strains *in vitro* will be reflected *in vivo*. The simulations show that a single-dose exposure to DHA would reduce the parasite load to a much smaller degree in the K13 mutant than in the revertant strain, leading to an ∼20-fold greater parasite burden ∼7 h after treatment (Fig. 4, top panel). There is no further substantial parasite killing at times longer than this, as the *in vivo* DHA concentrations become sublethal. As a result, a substantial fraction of the early ring population of the resistant Cam3.II strain (∼20%) (Fig. 4, top panel) is able to survive a single-dose DHA exposure, which helps to explain the *in vivo* resistance phenotype. The simulations also show that, as with the trends observed *in vitro*, the times taken to reduce the parasite load by 50% are similar for OZ277 and DHA treatments but somewhat longer for OZ439 treatment (up to ∼5 h for resistant strains). In the case of a short-lived drug, such as DHA (∼1-h half-life), such an increase in the time taken to kill 50% of parasites would present a major disadvantage, further increasing the risk of treatment failure in a resistant infection. However, the much longer *in vivo* half-life of OZ439 (see Table S2) means that the *in vivo* drug concentration is maintained for >72 h at a level much greater than the LC_50_ value observed in a standard kill assay (Table 1). This means that unlike the situation for DHA, where the drug is essentially depleted after 9 h, OZ439 will continue to efficiently kill both sensitive and resistant parasites well beyond 9 h, with anticipated activity for more than 72 h. (This is evident from Fig. 3, where a comparison is made of the loss of viability for parasites exposed to OZ439 for >48 h [black symbols] and the data for parasites exposed to DHA for 3 h [blue symbols] and 6 h [red symbols].) Thus, our analysis suggests that a single-dose exposure to OZ439 will reduce the parasite load in a resistant infection much more efficiently than a single dose of DHA.

### The activities of DHA and the ozonides are antagonized by a hemoglobinase inhibitor.

**FIG 5:**
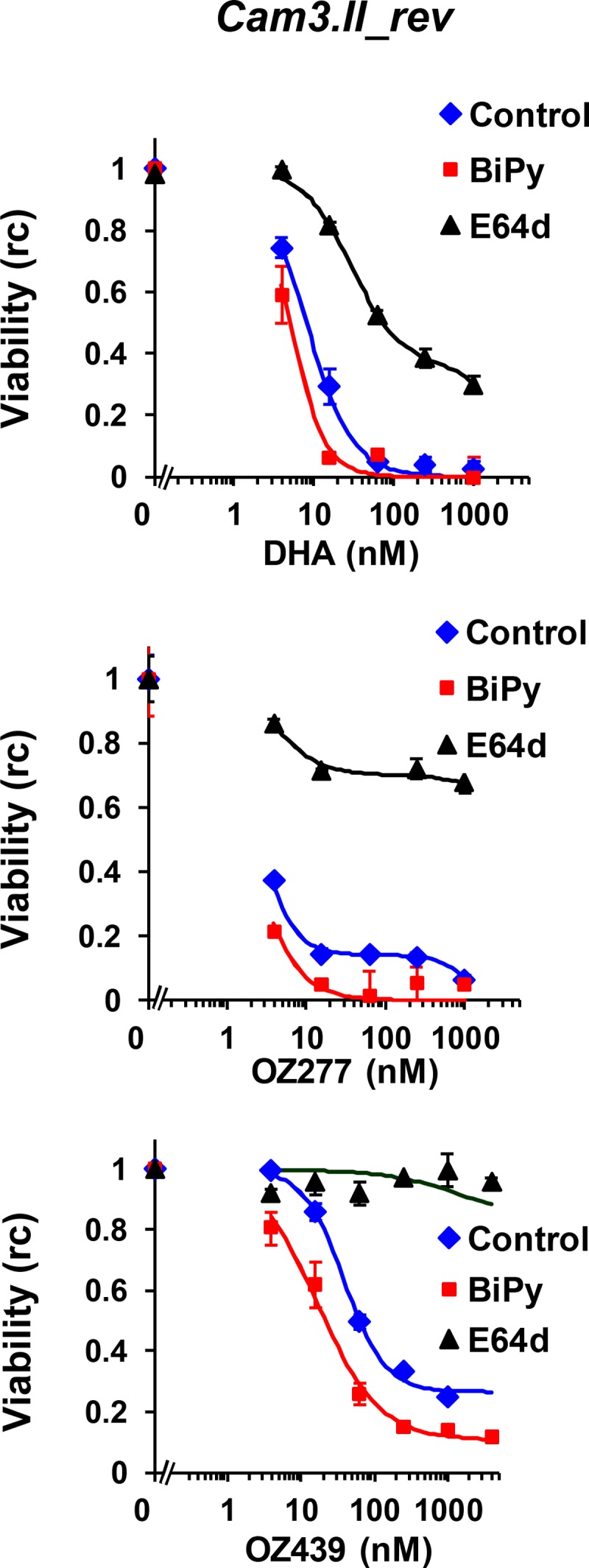
Role of hemoglobin degradation in the action of ozonides against K13 revertant parasites. Tightly synchronized early-ring-stage Cam3.II_rev parasites (1 to 1.5% parasitemia) were preincubated in the absence or presence of a sublethal concentration (determined by an initial analysis of the cytotoxicity of the compounds alone) of E64d (5 μM) or BiPy (150 μM) for 1 h before exposure to serial dilutions of DHA, OZ277, or OZ439 for 3 h. The drugs were removed using the enhanced washing protocol, and parasite viability (rc) was determined after >48 h. Data represent a typical set of data from two independent experiments, each performed in duplicate.

The activation of artemisinins via a reduced iron source is integral to their mode of action (34, 41). Recent work indicates that in the case of DHA, this iron source is heme generated during hemoglobin digestion, even when artemisinins are applied at the very early ring stage (34, 37). Accordingly, the falcipain inhibitor E64d exhibited significant antagonism of the action of a 3-h pulse of DHA against K13 revertant (Cam3.II_rev) parasites (Fig. 5). Like that of DHA, the activity of the ozonides was strongly antagonized by E64d (Fig. 5), indicating that their activity is dependent on hemoglobin degradation. In contrast, the iron chelator BiPy slightly synergized the activities of DHA and the ozonides, indicating that free iron makes little contribution to activation of the endoperoxides in very early rings (Fig. 5). Similar interactions were observed when E64d or BiPy was used in combination with a 6-h pulse of DHA or the ozonides against mid-ring-stage 3D7 parasites (see Fig. S3 in the supplemental material).

## DISCUSSION

In this work, we used a pulsed exposure assay to examine the activity of ozonides at different parasite stages. To achieve reliable estimates of efficacy, it was essential to ensure that the drugs were efficiently removed by the washing procedures employed at the end of the exposure period. We found that significant levels of ozonides remained in the culture wells when a standard washing procedure was employed. This was particularly true for OZ439, the most lipophilic of the three compounds tested. While the adherent fraction is likely quite low (on the order of 1% at micromolar concentrations), killing is caused by the continued exposure of the culture to the remaining drug for the duration of the incubation (>48 h). This can result in overestimation of the efficacy, particularly for resistant strains subjected to short exposures, which need a high level of drug to effect killing. This is important because it could result in the mischaracterization of resistant parasites as sensitive.

Artemisinins are activated via a reduced iron source generated during hemoglobin digestion (34, 37). Recent work (42) confirmed earlier studies (43) suggesting that artemisinins exert their activity by alkylating multiple targets within the parasite. Like the artemisinins, the ozonide antimalarials harbor an endoperoxide bridge; however, it is still unclear whether they are activated in the same way and are subject to the same mechanisms of resistance.

In this work, we showed that, as for DHA, short pulses of ozonides are more active against the trophozoite stage than against the mid-ring stage of 3D7 parasites, consistent with an enhanced hemoglobin degradation flux. Moreover, we found that falcipain inhibitors markedly antagonized the activity of the ozonides, as would be anticipated upon disruption of the supply of heme. In contrast, the iron chelator BiPy had very little effect under the conditions of our experiments, indicating that, like artemisinins (37), the ozonides are dependent on hemoglobin-derived heme rather than free iron for activation.

There is evidence that the parasite mounts a proteostasis response that counteracts the artemisinin-induced insult and that killing is achieved only when this defense system is overwhelmed (35). As a result, the extent of killing is highly dependent on the duration of drug exposure (34, 35, 37). In this work, we found that the killing of parasites by both DHA and the ozonides is highly nonlinear with respect to exposure time. For example, mid-ring-stage 3D7 parasites are insensitive to a short (3 h) exposure to drugs, but exposure to double that time (6 h) results in 100% killing, with an LC_50_ value that is not much higher than that observed for a 9-h pulse.

We demonstrated previously that in order for artemisinins to render parasites nonviable, the parasites need to be exposed to a sufficient concentration of activated endoperoxide for a sufficient time (34). This manifests as a delay following the application of endoperoxides before 50% of parasites are rendered nonviable. This delay is stage and strain dependent. The CED model predicts that killing will be more efficient if the endoperoxide is more efficiently activated (e.g., due to a higher level of activator or a more readily activated endoperoxide) (37). This is responsible for the particular sensitivity of trophozoite-stage parasites to endoperoxides (34).

The extent of killing will also be determined in part by stage- and strain-dependent differences in the ability of the parasite to deal with damaged proteins (34, 35, 37). This is expected to manifest as a change in the *t*^_50_ (time required to render 50% of parasites nonviable at a saturating drug concentration). For example, the very early rings of strains with wild-type K13 are particularly sensitive to DHA, likely because their cellular defense systems are particularly weak (35, 44). Similarly, the early rings of K13 mutant strains are less sensitive to DHA than those of wild-type strains, likely because K13 mutants exhibit an enhanced cellular defense system (35, 44). In this work, we showed that the differential sensitivity of different stages and strains to DHA was matched by similar differences in sensitivity to the ozonides, consistent with the suggestion that the basic mechanism of action of ozonides is similar to that of DHA and that they are prone to the same resistance mechanisms.

We found that the *K_M_* and *t*^_50_ values varied substantively depending on the chemical features of the endoperoxide, presumably due to differences in the efficiency of activation and/or the toxicity of the activated species. Access to the peroxide bond is more sterically restricted in OZ439 than in DHA (17, 18). We found that OZ439 exhibits moderately larger *t*^_50_ values than those of DHA, leading to lower levels of parasite inactivation when the exposure time is short. One possible interpretation is that the higher stability of OZ439 results in slower heme-mediated activation in the parasite, requiring a longer exposure time to render parasites nonviable. It is also possible that differences in the physicochemical properties of OZ439 may slow the rate of accumulation of the drug in the parasite or lead to different protein target interactions. A combination of these factors may be responsible for the requirement for a slightly longer exposure time to render 50% of parasites nonviable.

In an effort to understand the potential *in vivo* consequence of the *in vitro* behavior of the ozonides, we used our estimates of drug pharmacodynamics (CED model parameters) to predict parasite behavior in a patient. Parasite viability was simulated for the first 9 h following a single drug dose administered at the very early ring stage to patients harboring wild-type or K13 mutant parasites. We did not attempt to extend the simulation beyond 9 h because stage-dependent differences in sensitivity complicate predictions of the behavior of surviving parasites at longer time points (35). We employed the available pharmacokinetic profiles for the different drugs in patients or volunteers to represent the drug concentrations likely experienced by the parasites. Our simulations indicate that following drug exposure, reduction of parasite numbers to 50% of initial levels may take 1 to 2 h longer when OZ439 is employed than when DHA is used. Indeed, in a retrospective analysis of phase II clinical data, OZ439 was assessed for efficacy in patients with K13 mutant and wild-type parasites (25). In that study, a trend toward an extension of the clearance half-life was observed for patients infected with K13 mutant compared with wild-type parasites (5.5 h versus 4.4 h), although the results were not statistically significant.

Despite the predicted lower initial levels of killing of very early rings, the longer *in vivo* half-life of OZ439 means that it will perform much more effectively than OZ277 or DHA at reducing parasite burdens in clinical infections, particularly in the case of K13 mutant parasites. Because the *in vivo* concentrations of OZ439 are maintained well above the LC_50_ >48 h_ value for more than a full life cycle, K13 mutant parasites that survive exposure at the very early ring stage will continue to be exposed to OZ439 in the more sensitive trophozoite stage. Thus, our work supports clinical trials that are under way to assess OZ439 for use (in combination with an effective partner drug) as a single-dose treatment and for multidose treatment of infections in areas with a high penetrance of K13 mutations.

Clearly, there are a number of factors that complicate comparisons of the activities of different drugs *in vitro*, as well as attempts to extrapolate from *in vitro* to *in vivo* behavior. In particular, the differences in physicochemical properties between the three compounds suggest likely differences in protein binding both in the *in vitro* culture medium and *in vivo* in blood and plasma, leading to differences in unbound concentrations for the same total drug concentration. Since it is generally assumed that only unbound drug is available for permeating cell membranes and interacting with biological targets (45), these differences would be expected to affect the *in vitro* and *in vivo* activities. It also remains to be determined whether the ozonides induce dormancy in a subpopulation of parasites, as has been reported for the artemisinins (46, 47). Given these caveats, it will be critical to validate the predictions from our simulation by using detailed measurements of parasite killing and clearance times *in vivo*.

In summary, our data provide information about the mode of activation of the ozonides and show that a parasite harboring a K13 mutation exhibits reduced sensitivity to the ozonides. Despite this reduced sensitivity, the data strongly suggest that the longer half-life of OZ439 will result in significantly improved efficacy against K13 mutant parasites and that OZ439 will prove valuable in global efforts to battle artemisinin-resistant malaria. We anticipate that predictions of *in vivo* efficacy provided by our CED model will help to inform decisions about clinical use of the new endoperoxides.

## Supplementary Material

Supplemental material
