# Supplementary material for: Comparison of the Exposure Time Dependence of the Activities of Synthetic Ozonide Antimalarials and Dihydroartemisinin against K13 Wild-Type and Mutant Plasmodium falciparum Strains
Source: Antimicrob Agents Chemother. 2016 Jul 22;60(8):4501–10. doi: 10.1128/AAC.00574-16 (PMC4958167; doi:10.1128/AAC.00574-16)
Supplement: Supplemental material [file supp_60_8_4501__index.html]

Comparison of the Exposure Time Dependence of the Activities of Synthetic Ozonide Antimalarials and Dihydroartemisinin against K13 Wild-Type and Mutant Plasmodium falciparum Strains — Supplemental material 

# Comparison of the Exposure Time Dependence of the Activities of Synthetic Ozonide Antimalarials and Dihydroartemisinin against K13 Wild-Type and Mutant Plasmodium falciparum Strains

## Supplemental material

- Supplemental file 1 -

  Fig. S1-S3 and Tables S1 and S2

  PDF, 246K
