# Supplementary material for: Comparison of the Exposure Time Dependence of the Activities of Synthetic Ozonide Antimalarials and Dihydroartemisinin against K13 Wild-Type and Mutant Plasmodium falciparum Strains
Source: Antimicrob Agents Chemother. 2016 Jul 22;60(8):4501–10. doi: 10.1128/AAC.00574-16 (PMC4958167; doi:10.1128/AAC.00574-16)
Supplement: Supplemental material [file AAC.00574-16_zac008165346so1.pdf]

Antimicrobial Agents and Chemotherapy

Supplementary Material

A comparison of the exposure time-dependence of the activities of synthetic ozonide antimalarials and dihydroartemisinin against K13 wildtype and mutant *Plasmodium falciparum*

Tuo Yang, Stanley C. Xie, Pengxing Cao, Carlo Giannangelo, James McCaw, Darren J. Creek, Susan A. Charman, Nectarios Klonis and Leann Tilley

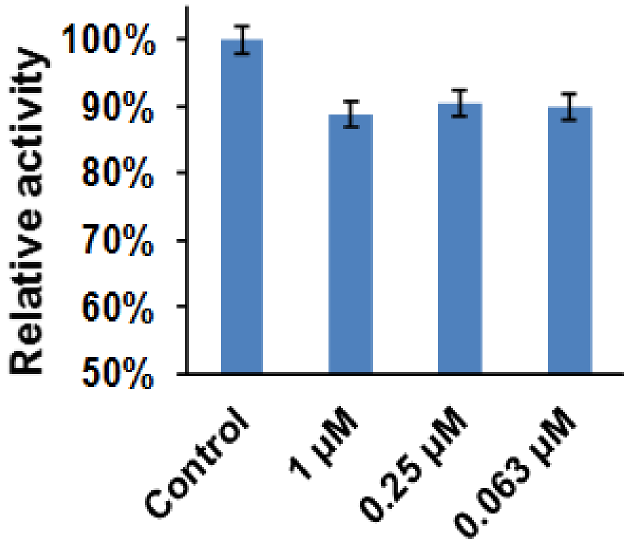

Figure S1. **Loss of drugs from the supernatant**

The indicated drug concentrations were added to complete medium in the presence of red blood cells (RBCs; 0.2% hematocrit), then transferred to new v-bottomed wells and serially diluted. Drug activity is expressed relative to a control which was not subject to the transfer. Error bars reflect the range from the average of two independent experiments each performed in duplicate or triplicate.

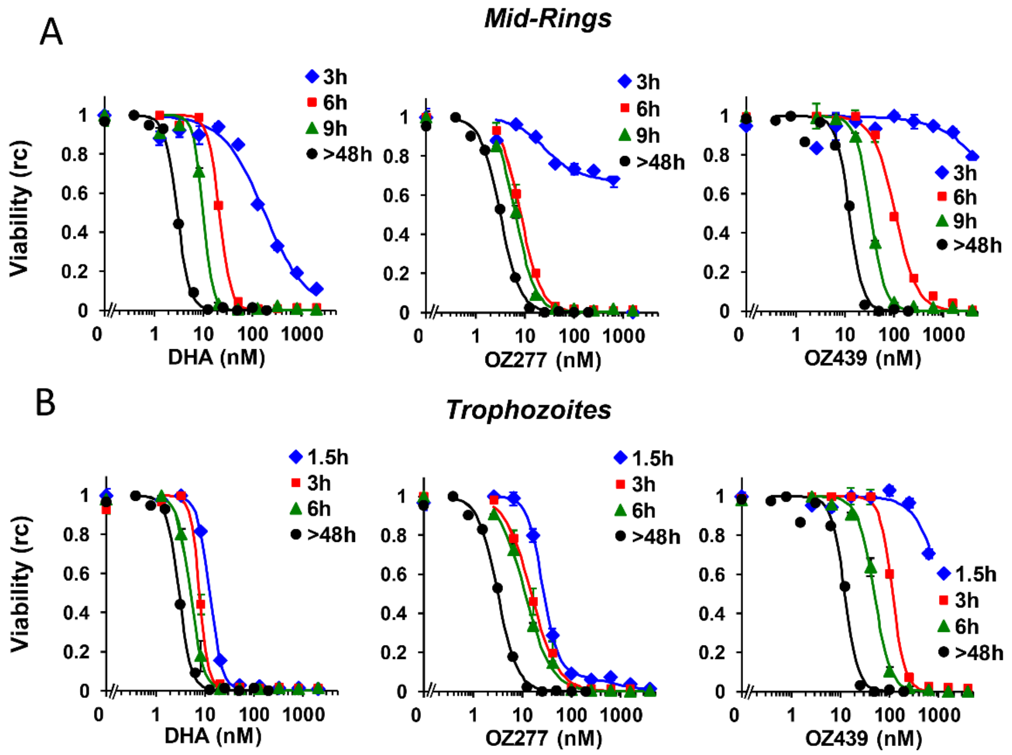

Figure S2. Effect of pulse duration of different endoperoxides on viability (replication competence) of 3D7 ring and trophozoite stage parasites

Mid-ring (A) and trophozoite (B) stage 3D7 parasites were incubated with drugs for the pulsed exposures indicated or the drug pressure was maintained for >48 h (black symbols). For pulse assays, the drugs were removed using the enhanced washing protocols and parasite viability (rc, replication competence) was determined after >48 h. Data shown are representative of experimental replicates. Error bars correspond to the range of technical replicates from the individual experiments.

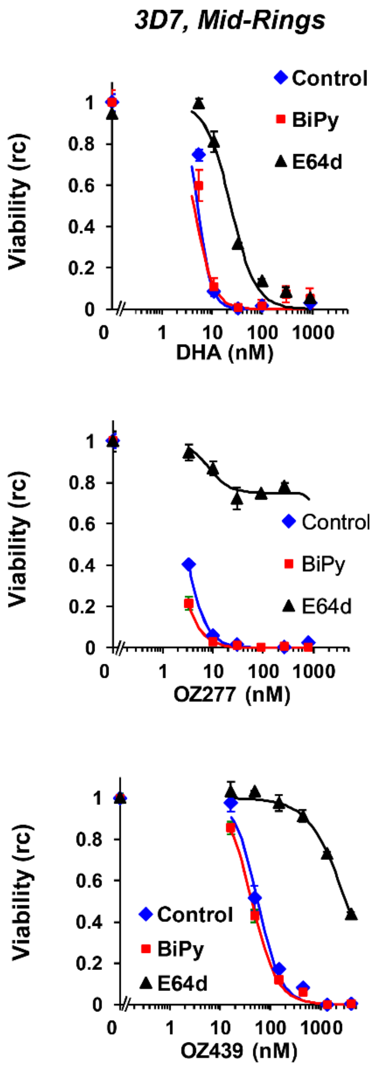

Figure S3. Ozonide activity against mid-ring 3D7 is antagonized by a hemoglobinase inhibitor

Synchronized 3D7 parasites (1-1.5% parasitemia; mid-ring) were pre-incubated in the absence or presence of a sub-lethal concentration (determined by an initial analysis of the cytotoxicity of the compounds alone) of E64d (10 μM) or BiPy (1 mM) for 1 h before exposure to serial dilutions of DHA, OZ277 or OZ439 for 3 h. The drugs were removed using the enhanced washing protocols and parasite viability (rc, replication competence) was determined after >48 h. Data are from a typical experiment performed in duplicate and are representative of data from two experiments.

Table S1. **Potency of DHA and ozonides against different *P. falciparum* strains in pulsed drug exposure assays**

Early ring stage K13 mutant (Cam3.II) or wildtype (Cam3.II_rev) cultures were incubated for 3, 6 or 9 h with the indicated drugs. Following application of the enhanced washing protocol to remove drug, the samples were cultured for >48 h prior to determination of viability (replication competence).

	**DHA**		**OZ277**		**OZ439**	
	**Cam3.II_rev**	**Cam3.II**	**Cam3.II_rev**	**Cam3.II**	**Cam3.II_rev**	**Cam3.II**
LC_{50_3h} (nM) [a]	12 ± 5	>700	6 ± 3	>700	200 ± 100	>700
LC_{50_6h} (nM) [a]	10 ± 6	18 ± 7	8 ± 6	14 ± 9	45 ± 16	390 ± 160
LC_{50_9h} (nM) [b]	4	5 ± 2	3 ± 2	5 ± 2	27 ± 15	60 ± 30

	**DHA**		**OZ277**		**OZ439**	
	**Cam3.II_rev**	**Cam3.II**	**Cam3.II_rev**	**Cam3.II**	**Cam3.II_rev**	**Cam3.II**
V_{min_3h} [a]	0.06 ± 0.03	0.50 ± 0.03	0.15 ± 0.05	0.53 ± 0.08	0.30 ± 0.05	0.64 ± 0.03
V_{min_6h} [a]	0.03 ± 0.01	0.32 ± 0.02	0.04 ± 0.03	0.45 ± 0.05	0.07 ± 0.02	0.46 ± 0.03
V_{min_9h} [b]	0.01	0.11	0	0.21 ± 0.01	0.03 ± 0.01	0.22 ± 0.01

[a] Data represent the mean ± S.D. of data from three independent experiments, each with two technical replicates.

[b] Data represent the mean ± range of data from two independent experiments, each with two technical replicates.

Table S2. **Drug pharmacokinetic parameters used for simulations of drug activity *in vivo***

	**DHA**[a]	**OZ277**[b]	**OZ439**[c]
Dose[d]	2 mg/kg	200 mg	800 mg
t_{max} (h)[e]	1.0	5.0	3.0
C_{max}(nM)[f]	2820	300	1700
$f_{(1)}$[g]	1	1	0.95
t½ $_{(1)}$ (h)[h]	0.9	3.5	11.0
t½ $_{(2)}$ (h)[i]	-	-	66

[a] Pharmacokinetics measured in malaria patients from Pailin. Data derived from (1). Note that the dose was administered as artesunate but that the pharmacokinetic parameters are for DHA.

[b] Pharmacokinetics correspond to 200 mg administered to healthy fasted patients. Data derived from (2).

[c] Pharmacokinetics correspond to 800 mg oral dispersion administered to healthy fasted patients. Data derived from (3).

[d] The administered dose which corresponds to the pharmacokinetic parameters shown in this Table.

[e] Time for drug to reach maximum concentration *in vivo* (C_{max})

[f] The maximum drug concentration *in vivo*

[g] The fraction of the drug decay that can be attributed to the first component, for a drug that exhibits bi-exponential decay. The decay of OZ439 was assumed to be bi-exponential based on data from (3). A value of 1 denotes mono-exponential decay of the drug.

[h] Drug half-life ascribed to the first component (1).

[i] Drug half-life ascribed to the second component (2).

References

1. **Dondorp AM, Nosten F, Yi P, Das D, Phyo AP, Tarning J, Lwin KM, Ariey F, Hanpithakpong W, Lee SJ, Ringwald P, Silamut K, Imwong M, Chotivanich K, Lim P, Herdman T, An SS, Yeung S, Singhasivanon P, Day NP, Lindegardh N, Socheat D, White NJ.** 2009. Artemisinin resistance in *Plasmodium falciparum* malaria. N Engl J Med **361:**455-467.
2. **Saha N, Moehrle JJ, Zutshi A, Sharma P, Kaur P, Iyer SS.** 2014. Safety, tolerability and pharmacokinetic profile of single and multiple oral doses of arterolane (RBx11160) maleate in healthy subjects. J Clin Pharmacol **54:**386-393.
3. **Moehrle JJ, Duparc S, Siethoff C, van Giersbergen PL, Craft JC, Arbe-Barnes S, Charman SA, Gutierrez M, Wittlin S, Vennerstrom JL.** 2013. First-in-man safety and pharmacokinetics of synthetic ozonide OZ439 demonstrates an improved exposure profile relative to other peroxide antimalarials. Br J Clin Pharmacol **75:**524-537.
